# Community-Based Physical Activity Interventions for Treatment of Type 2 Diabetes: A Systematic Review with Meta-Analysis

**DOI:** 10.3389/fendo.2013.00003

**Published:** 2013-01-29

**Authors:** Ronald C. Plotnikoff, Sarah A. Costigan, Nandini D. Karunamuni, David R. Lubans

**Affiliations:** ^1^Priority Research Centre in Physical Activity and Nutrition, University of NewcastleCallaghan, NSW Australia; ^2^Faculty of Physical Education and Recreation, University of AlbertaEdmonton, AB, Canada

**Keywords:** type two diabetes, physical activity, community-based intervention, treatment, HbA1c

## Abstract

Evidence suggests engaging in regular physical activity (PA) can have beneficial outcomes for adults with type 2 diabetes (TD2), including weight loss, reduction of medication usage and improvements in hemoglobin A1c (HbA1c)/fasting glucose. While a number of clinical-based PA interventions exist, community-based approaches are limited. The objective of this study is to conduct a systematic review with meta-analysis to assess the effectiveness of community-based PA interventions for the treatment of TD2 in adult populations. A search of peer-reviewed publications from 2002 to June 2012 was conducted across several electronic databases to identify interventions evaluated in community settings. Twenty-two studies were identified, and 11 studies reporting HbA1c as an outcome measure were pooled in the meta-analysis. Risk of bias assessment was also conducted. The findings demonstrate community-based PA interventions can be effective in producing increases in PA. Meta-analysis revealed a lowering of HbA1c levels by −0.32% [95% CI −0.65, 0.01], which approached statistical significance (*p* < 0.06). Our findings can guide future PA community-based interventions in adult populations diagnosed with TD2.

## Introduction

The estimated global prevalence of diabetes in 2010 was 6.4% (equating to ∼285 million adults). Type two diabetes (T2D) has contributed to the majority of these cases (Shaw et al., 2010). T2D is largely related to weight gain associated with a combination of low physical activity (PA) levels and a consumption of an energy dense diet (Nolan et al., 2011). Evidence suggests engaging in regular PA can have beneficial outcomes for adults with T2D (Sigal et al., 2007), including improved self-management of T2D, weight loss, increased fitness, reduction of medication usage and improvements in HbA1c/fasting glucose (Church et al., 2010).

Researchers have investigated a variety of PA intervention strategies to encourage individuals with T2D to be more active. One such strategy is supervised facility-based exercise training, which has potential to improve glycemic control and other cardiovascular risk factors (Sigal et al., 2007; Balducci et al., 2010; Church et al., 2010). However, these programs are often resource-intensive, only available in metropolitan areas, and their long-term sustainability is indeterminate.

Other strategies to promote PA in T2D adults include individual-based approaches such as medication use and behavior change. These strategies have become increasingly common, with information on diet, exercise, and medication generally provided by health care professionals (e.g., GP, pharmacist, specialist). However, encouraging individuals with T2D to adopt behavior modification during short visits to their GP, is challenging. These self-management approaches also have modest efficacy in the short term, and long-term assessments are often very limited (Plotnikoff, 2006). Further, these types of interventions can be “out of reach” for individuals with a low-income, low education, lack of access to care, and cultural and linguistic barriers.

On the other hand, community-based approaches may help improve self-management of T2D by addressing barriers encountered in both facility-based approaches and individual-based approaches. For example, community-based interventions can deliver culturally appropriate health education which can improve self-care compliance and adherence to self-management practices (Two Feathers et al., 2005; Vachon et al., 2007). Further, community-based interventions can be more cost-effective and practical, may have better long-term effectiveness, and the potential to reach a large proportion of individuals who are in most need of treatment (Two Feathers et al., 2005; Plotnikoff, 2006; Vachon et al., 2007).

The objective of this paper is to conduct a systematic review with meta-analysis to assess the effectiveness of community-based, PA interventions for the treatment of T2D in adult populations. The meta-analysis focused on interventions that assessed changes in hemoglobin A1c (HbA1c) as an outcome measure. This review includes interventions that employ community-based approaches (e.g., community centers, local facilities, community-based educators), rather than those delivered in workplace or traditional clinical settings. To the best of our knowledge, this is the first systematic review to examine the efficacy of community-based PA approaches for treatment of T2D in adults.

## Methods

### Search strategy and data sources

Studies published in peer-reviewed journals were identified by two authors (SAC & NK) through a structured electronic database from January 2002 until June 2012 in CINAHL, Web of Science, Scopus, and Medline. The following search strings were used: (PA OR exercise) AND (Type two diabetes OR Type 2 Diabetes OR T2D) AND (Intervention OR Program) AND Community. These strings were further limited to subjects 18+ years and English language. In the first stage of the literature search, titles, and abstracts of identified articles were checked for relevance and additional articles known to the authors were assessed for possible inclusion. In the second stage, full-text articles were retrieved and considered for inclusion. In the final stage, the reference lists of retrieved full-text articles were searched for additional studies.

### Study selection criteria

Quantitative community-based PA interventions for treatment of T2D were included in this review. Interventional studies were considered for inclusion. Studies were eligible for inclusion if they: (1) included participants 18+ years; (2) employed a PA based intervention [i.e., studies were classified by proportion of the intervention which was PA based (i.e., ≥50%)]; (3) employed community-based approaches (e.g., community centers, local facilities, community-based educators; (4) implemented strategies for treating existing cases of T2D (e.g., increased PA, weight loss, reduction of medication usage, improvements in HbA1c); and (5) were quantitative studies.

Studies not included in this review consist of publications which: (1) examined children or adolescents (aged < 18 years); (2) were non-interventional; (3) had an intervention that was not at least 50% focused on PA; (4) did not include strategy for < 50% of the total intervention; (5) were conducted in traditional clinical settings; (6) employed preventative measures to adults at risk of T2D (i.e., cases of pre-diabetes); (7) used qualitative studies; and (8) were conference abstracts, dissertations, theses, and articles published in non-peer-reviewed journals.

### Data extraction

Initially, articles were assessed for eligibility by a single reviewer (SAC) based on the study title. After this initial cull, study abstracts were assessed by two authors (SAC & NK) independently in an unblinded standardized manner. Findings were compared and differences were resolved by consensus or by a third author (RCP). Specific study characteristics were identified and extracted by two authors (SAC & NK). These characteristics included the country of origin, study-design, size/source of study population, details of the PA intervention and community components of the study (see Table 1).

**Table 1:** **Study characteristics**.

Author (date)	Study design	*N*	*N* per group	Recruitment	Intervention length	Community based intervention component	PA aspect	Primary Outcome(s)	Results
Plotnikoff et al. (2011), Canada	RCT	96	*N*(Con) = 49 *N*(Int) = 47	Diabetes clinics	16 weeks	Recruitment Use of community-based facilities	Structured resistant training program	HbA1c PA (METmins) Fitness (mCAFT)	*HbA1c* [baseline mean (SD)] Control = 7.8%(2.0); 61.75 mmol/mol Intervention = 7.3% (1.3); 56.28 mmol/mol *HbA1c* [12-months mean (SD)] Control = 7.2%(1.6); 55.19 mmol/mol Intervention = 7.2%(1.4); 55.19 mmol/mol HbA1c mean change at 12 months Control group = −0.5; 95% CI: −0.9 to −0.2) Intervention group = −0.4; 95% CI: −0.7 to 0.0 *PA (METmin*) Mean change at 12 months Con: −33.9(CI: −213.6–145.8) Int: 654.2 (CI:466.9−841.6)** Fitness (mCAFT) Mean change at 12 months Con: −1.9(−16.3–12.5) Int: 28.2(14.2–42.2)**
Piette et al. (2011), USA	RCT	291	*N*(Con) = 146 *N*(Int) = 145	Community-based non-profit healthcare system; Additional patients were self-referred based on advertisements and newsletters	12 months	Recruitment Primary care providers identified for each participant	Pedometer-based walking program	HbA1c	*HbA1c* [baseline mean (SD)] Control = 7.7%(1.7); 60.66 mmol/mol Intervention = 7.5%(1.7); 58.47 mmol/mol *HbA1c* [12-months mean (SD)] Control = 7.7%(1.7); 60.66 mmol/mol Intervention = 7.7%(1.8); 60.66 mmol/mol HbA1c Between-group Difference (95% CI) = 0.07 (−0.26 to 0.40)
Kruse et al. (2010), USA	RCT	79	*N*(Con) = 38 *N*(Int) = 41	Program open to anyone meeting the criteria	12 months	Recruitment Community setting	Individual sessions with physical therapist focusing on leg strength and promoting balance Weekly 1-h sessions to improve leg strength and balance Adding 100 steps to their daily activities every 2 weeks	Strength and balance BMI Falls	No significant differences between control and intervention groups for the primary outcomes.
Less et al. (2009), Jamaica	Prospective cohort study	293	*N*(Con) = 135 *N*(Int) = 158	Health centers	6 months	Recruitment Community setting	Physical activity log, they tracked their success in achieving the goal of 30-min physical activity each day	HbA1c	*HbA1c* (Baseline Mean) Control = 8.03%; 64.26 mmol/mol Intervention = 7.95%; 63.39 mmol/mol *HbA1c* (Post Mean) Control = 8.62%; 70.71 mmol/mol Intervention = 7.32%**; 56.50 mmol/mol
Martyn-Nemeth et al. (2010), USA	Pretest/post-test design	16	One group *N* = 16	Community-based clinic that provides care for low-income, independent-living community residents.	12 weeks	Input provided from: community-based nurses diabetic nurse educator medical staff exercise physiologist	Regular low-impact exercise routine led by nurses Weekly exercise sessions were led for 12 weeks 60-min, low-impact exercise routine	HbA1c Lipid panel BMI	No significant differences between control and intervention groups for the primary outcomes.
Mathieu et al. (2008), Canada	RCT	58	*N*(Con) = 25 *N*(Int) = 33	Kinesiologists in community-based facilities were responsible for recruiting participants	10 week	Recruitment Group training sessions	DiabetAction program introduced participants to cardiovascular, resistance, balance, and flexibility exercises 10 group sessions consisting of a 60-min PA period of aerobic exercisesat light to moderate-intensity, resistance exercises, and balance and flexibility exercises	PA	No significant differences between control and intervention groups for the primary outcomes
Taylor et al. (2009), USA	RCT	24	*N*(Con) = 13 *N*(Int) = 11	Local community	2 months	Recruitment Use of community fitness center facilities	Physical therapist– directed exercise counseling Fitness center–based exercise training 4 sets of up to 8 repetitions of each exercise using 80% of the 1-repetition maximum (1-RM) on 2 non-consecutive days per week for 2 months	Muscular strength Exercise capacity	No significant differences between control and intervention groups for the primary outcomes
Skoro-Kondza et al. (2009), UK	RCT	59	*N*(Con) = 30 *N*(Int) = 29	GP’s	12 weeks	Community-based sessions	Regular yoga classes 24 × 90-min yoga classes over 12 weeks	HbA1c	*HbA1c* Control group: Baseline mean = 7.03%; 53.33 mmol/mol Mean at 6 months = 7.30%; 56.28 mmol/mol (mean change −0.28; 95% CI −0.10–0.66) Intervention group: Baseline mean = 7.06%; 53.66 mmol/mol Mean at 6 months = 7.01%; 53.11 mmol/mol (mean change −0.05; 95% CI −0.26–0.16).
Dutton et al. (2008), USA	RCT	85	*N*(Con) = 39 *N*(Int) = 46	Community diabetic clinic	4 weeks	Recruitment	Stage-targeted booklet addressing: Motivation Self-efficacy Goal-setting Social support Problem-solving Two-page letter tailored to their: Stage of change Self-efficacy Pros/cons for activity Current activity levels/preferences Activity goals	PA	*Weekly minutes of PA* Control group: Baseline mean = 107.3 (102.2) Mean change from baseline to post-intervention = −1.9, 95% CI = −37.0 to 33.2 Intervention group: Baseline mean = 148.5 (121.3) Mean change from baseline to post-intervention = 21.8, 95% CI = −16.3 to 59.9
Klug et al. (2008), USA	Non-randomized one group before and after design	243	One group At 4 months *N* = 147	Announcements and presentations to internal and external community groups, posted fliers, placed bulletins in newsletters, contacted eligible older adults directly, depended on “word of mouth,” worked with local media, and partnered with other agencies	6 months	Recruitment Setting Peer leader	Weekly support sessions for PA and diet	Self-efficacy Self-rated health	Self-efficacy and self-rated health scores significantly improved
Speer et al. (2008), USA	Convenience sample	260	One group *N* = 144	Seniors centers	4 months	Recruitment Experts from the local university	Physical activity incorporated into sessions 40–60 min weekly sessions	HbA1c	HbA1c Baseline 7.00% (SD = 1.39); 53.01 mmol/mol Post-intervention 6.7% (SD = 1.17)*; 50.38 mmol/mol
Brooks et al. (2007), USA	RCT	62	*N*(Con) = 31 *N*(Int) = 31	Not reported	16 weeks	Exercise training conducted in community setting	Regular exercise and strength training	HbA1c Body composition Muscle strength Muscle quality 3x/week for 16 weeks for exercise training. Sessions included 35-min strength training 3 sets of 8 repetitions on each machine Training intensity during wks 1–8 were 60–80% of baseline 1-repetition maximum (1-RM), and during wks 10–14 were 70–80% of mid-study 1-RM	*HbA1c* [baseline mean (SE)] Control = 7.8% (1.6); 61.75 mmol/mol Intervention = 8.7% (1.8); 71.58 mmol/mol HbA1c (post) Control = 8.3%(1.3); 67.21 mmol/mol Intervention = 7.6%(1.5)**; 59.56 mmol/mol Control group: Mean change from baseline to post-intervention [mean (SE)] = 0.4 (0.3) Intervention group: Mean change from baseline to post-intervention [mean (SE)] = −1.0 (0.2) Significant change score differences for the intervention group vs. controls on upper body muscle strength, lower body muscle strength, Whole-body lean tissue mass, Muscle quality, Type 1 and Type 2 muscle fire area
Dunstan et al. (2006), Australia	RCT	57	*N*(home training) = 26 *N*(center training) = 27	Recruited from the clinics of the International Diabetes Institute and by a local media campaign.	12 months	Community fitness center facilities used	Resistance training program 2 days/week Low-intensity stationary cycling plus stretching exercises and 45 min of high intensity resistance training Goal was to achieve between 75 and 85% of the current 1-RM. Three sets of eight repetitions were performed for all exercises	HbA1c	HbA1c Home group: Baseline mean = 7.5%(0.5); 58.47 mmol/mol Center group: Baseline mean = 7.8%(0.9); 61.75 mmol/mol Home group: Mean change from baseline: M(SD): −0.1(1.1) Center group: Mean change from baseline: M(SD): −0.4(1. 0)**
Boyd et al. (2006), USA	Non-randomized one group before and after design	48	One group *N* = 35	Recruited and referred by physicians, physicians assistants, advance nurse practitioners	12 months	Partnership between a community health centers to improve access to exercise for low-income patients with type 2 diabetes	3 month YMCA membership exercise classes twice per week lasting up to 12 months	HbA1c Blood pressure LDL Weight	HbA1c Baseline: 8.6%; 70.49 mmol/mol Post-intervention: 6.9%*; 51.91 mmol/mol *Blood pressure* Baseline systolic:130 mmHg Post-intervention:126 mmHg Baseline diastolic:79 mmHg Post-intervention diastolic: 75 mmHg* LDL Baseline: 116 mg/dL Post-intervention: 99 mg/dL* HDL Baseline: 41 mg/dL Post-intervention: 44 mg/dL *Weight* Baseline: 218 lb Post-intervention: 206 lb
Engel and Lindner (2006), Australia	RCT	57	*N*(coaching only) = 28 *N*(Pedometer) = 22	Local media campaign	6 months	Recruitment Group coaching sessions	Group coaching sessions Pedometer use values of between 6000 and 8500 steps per day (healthy older adults) and between 3500 and 5500 steps per day (older adults)	HbA1c Blood pressure BMI PA Fitness (shuttle run)	HbA1c Coaching only group: Baseline mean = 7.70%(1.39); 60.66 mmol/mol Mean change from baseline to post-intervention = −0.2(1.1) Pedometer group: Baseline mean = 7.14%(0.98); 54.54 mmol/mol Mean change from baseline to post-intervention = 0.02(1.0) *Systolic blood pressure* *Pedometer* Mean change from baseline = 2.4 (18.3) Coaching = –2.9 (14) *Diastolic blood pressure* Mean change from baseline Pedometer = 1.6 (9.9) Coaching = –4.1 (9.9) *BMI* Mean change from baseline Pedometer = –0.7 (2.3) Coaching = –0.7 (1.5) *Fitness* Mean change from baseline Pedometer = 65 (102) Coaching = 49 (72)
Two Feathers et al. (2005), USA	Non-randomized one group before and after design	91	One group *N* = 91	Health care systems in detroit	5 months	Locally based community resident meetings	Some meetings focused on increasing PA to improve patients self-management of diabetes	HbA1c	HbA1c Baseline: 8.4% (SD = 2.3); 68.31 mmol/mol Post-intervention: 7.6% (SD = 1.9)*; 59.56 mmol/mol
Brandon et al. (2003), USA	RCT	31	*N*(Con) = 15 *N*(Int) = 16	Veterans affairs diabetic clinic, a computerized research center database, local diabetic clinics, and senior centers, and by word of mouth	24 months	Recruitment Community PA facilities	Regular training for 24 months 3 sessions per week Strength training was completed on an 11-station Nautilus machine. The subjects completed 3 sets of 8–12 repetitions per exercise. The intensity was 50, 60, and 70% for sets 1, 2, and 3, respectively. Each 1-h session consisted of 50 min of strength-training exercises and 10 min of warm-up, flexibility, and cool-down exercises	Mobility Strength	*Strength-*hip flexors Control: 0.38(0.07) Intervention: 0.44(0.12) *Mobility*-timed up and go: Control: 8.3(1.0) Intervention: 7.6(1.8) There was a group × time effect as the Intervention group increased 31.4% (*p* < .001) in strength for all muscle groups after the first 6 months, and the strength gains were retained at 24 months. There was also a group × time effect for mobility as performance increased 8.6 and 9.8% (*p* < 032 and *p* < 0.031) for the first 6 and 12 months, respectively, but decreased to 4.6% above baseline at the end of the intervention.
Goldhaber-Fiebert et al. (2003), Costa Rica	RCT	75	*N*(Con) = 28 *N*(Int) = 33	Community GP’s	12 weeks	Intervention classes conducted by: Nutritionists Local volunteers/leaders	60 min walking group sessions three times per week for 12 weeks	HbA1c BMI Weight Fasting plasma glucose Serum lipids Blood pressure	HbA1c Control group: Baseline mean = 8.6%(3.9); 70.49 mmol/mol Mean change from baseline: −0.4(2.3) Intervention group: Baseline mean = 8.6%(3.7); 70.49 mmol/mol Mean change from baseline: −1.8(2.3)** Significant decreases in weight, BMI, and fasting plasma blood glucose for intervention vs. control group
Keyserling et al. (2002), USA	RCT	200	*N*(clinic and community) *b* = 54 *N*(clinic only) = 59 *N*(minimal intervention) = 58	Community GP’s	6 months	Phone calls to participants and group sessions	PA component developed to increase moderate-intensity PA to a cumulative total of 30 min a day	PA	PA (kcal/day) Clinic and community: 364 (SE:168.7) Clinic only: 322 (SE:207.1) Minimal intervention: 297 (SE:167.2) *Change scores over 24 months* Clinic and community vs. control: >44.1 (SE:15.8) [CI:13.1 to 75.1]** Clinic only vs Control: >33.1 (SE:15.1) [CI:3.3 to 62.8]**
Plotnikoff et al. (2012), Canada	RCT	287	*N*(Con) = 84 *N* (print materials) = 71 *N*(telephone counseling) = 73	General advertising strategies	12 months	Recruitment Phone calls to participants	PA guidelines Personalized print materials Telephone counseling Use of a pedometer	HbA1c PA	HbA1c [Baseline mean(SE) values] Control group = 7.08%(0.07); 53.88 mmol/mol Print materials = 7.08%(0.07); 53.88 mmol/mol Telephone counseling = 7.11%(0.07); 54.21 mmol/mol [12-months mean(SE) values] Control = 7.07%(0.07); 53.77 mmol/mol Print materials = 7.00%(0.08); 53.01 mmol/mol Telephone counseling = 7.28%(0.08); 56.07 mmol/mol Adjusted difference between-groups Print materials group – Control group:− 0.07 (95 % CI − 0.31 to 0.17) Telephone counseling group − Control group: 0.18 (95 % CI − 0.07 to 0.42) MVPA Control = 137.8 (SE:168.4) Print materials = 174.5 (SE:168.0) Telephone counseling = 153.8 (SE:166.6) *Steps* Control = 20,097 (SE:7313) Print materials = 20,821 (SE:7308) Telephone counseling = 21,493 (SE:7249)
Plotnikoff et al. (2010), Canada	RCT	48	*N*(Con) = 21 *N*(Int) = 27	Diabetes clinics	16 weeks	Recruitment Exercise program conducted in participants home	RT group performed regular exercise program Structured exercise program on 3 nonconsecutive days per week. Eight exercises were performed per session 16-week program Two sets of 10–12 repetitions at 50–60% of 1-RM	Muscle strength Glucose control	Muscle strength Bench press 1-RM (kg) Mean difference: 9.3 kg** (CI: 3.7, 15.0) Int>Con Control = 44.8(15.9) Intervention = 55.5(26.3) Leg press 1-RM (kg) Mean difference:41 kg** (CI: 15.1, 67.0) Int>Con Control = 93.8(63.4) Intervention = 175.0(141.5) Upright row 1-RM (kg) Mean difference: 12.4 kg** (CI: 7.3, 17.5) Int>Con Control = 56.6(20.6) Intervention = 69.8(27.8) HbA1c (baseline values) Control = 6.81%(0.83); 50.93 mmol/mol Intervention = 6.89%(1.50); 51.80 mmol/mol HbA1c (post-intervention values) Control = 6.77%(0.80); 50.49 mmol/mol Intervention = 6.97%(1.35); 52.68 mmol/mol Adjusted difference between-groups: (RT group – control group) = 0.3; 95% CI 0.1 to 0.4
Davies et al. (2008), UK	Cluster RCT	652	*N*(Con) = 248 *N*(Int) = 314	Community GP clinics	12 months	Delivered in the community	Group education program associated with benefits of PA	HbA1c	HbA1c Control group: Baseline mean = 7.9%(2.0); 62.84 mmol/mol Mean change from baseline to post-intervention = −1.21; 95% CI −1.40 to −1.02 Intervention group: Baseline mean = 8.3%(2.2); 67.21 mmol/mol Mean change from baseline to post-intervention = −1.49; 95% CI −1.69 to −1.29

*Con, control; Int, intervention; BMI, body mass index; GP, general practitioners; HbA1c, hemoglobin A1c; METmins, metabolic equivalent minutes; RCT, randomized control trial; MVPA, moderate to vigorous physical activity*.

*Note: Numbers in parenthesis () refer to SD unless otherwise stated*.

*Significant (*p* < 0.5) within group change (one group design).

**Significant (*p* < 0.5) between-group change (Intervention vs. Control).

### Synthesis of results

Meta-analysis was conducted for studies that reported their effect on HbA1c using RevMan version 5.1 [The Nordic Cochrane Centre, The Cochrane Collaboration, Copenhagen, Denmark; Review Manager (RevMan), 2011]. As recommended by the Cochrane Collaboration, post-test means (i.e., Less et al., 2009; Plotnikoff et al., 2010, 2012; Piette et al., 2011) or change scores (i.e., Goldhaber-Fiebert et al., 2003; Dunstan et al., 2006; Engel and Lindner, 2006; Brooks et al., 2007; Davies et al., 2008; Skoro-Kondza et al., 2009; Plotnikoff et al., 2011) and their SD were used in the analysis. One study compared multiple treatment groups with a single control group (*n* = 1), to avoid double counting, the sample size of the control group was divided by two. All data were considered continuous and therefore the mean difference (MD) with 95% confidence intervals was used to determine effect measures. Statistical heterogeneity was examined via Chi-squared and the *I^2^-*Index tests. A guide to the interpretation of heterogeneity based on the *I^2^-*Index is as follows: 0–40% might not be important; 30–60% may represent moderate heterogeneity; 50–90% may represent substantial heterogeneity; and 75–100% considerable heterogeneity (Deeks et al., 2008).

### Risk of bias

Risk of bias was assessed using a nine-item checklist tool adapted from the Consolidated Standards of Reporting Trials (CONSORT) statement and previous reviews (van Sluijs et al., 2007; Young et al., 2012). The following items were assessed: (1) baseline results reported separately for each group; (2) randomization clearly described and adequately completed; (3) dropout ≤ 20% for ≤6 months follow-up and ≤30% for >6 months follow-up; (4) assessor blinding; (5) intention-to-treat analysis; (6) confounders accounted for in analyses; (7) summary results presented and estimated effect sizes and precision estimates; (8) power calculation reported and study adequately powered; and (9) an objective measure of PA was used.

Each item was scored as “present” (✓), “absent” (×), or “unclear or inadequately described” (?). Depending on the study-design, some items were not applicable (n/a). Unweighted sum totals were calculated for each study using a predefined scoring system (✓ = 1 |× = 0 | ? = 0 | n/a = 0). Low risk of bias studies were regarded as those with a 8–9, a moderate risk of bias presented scores of 4–7, high risk of bias scored 0–3.

## Results

### Description of studies

**Figure 1:**
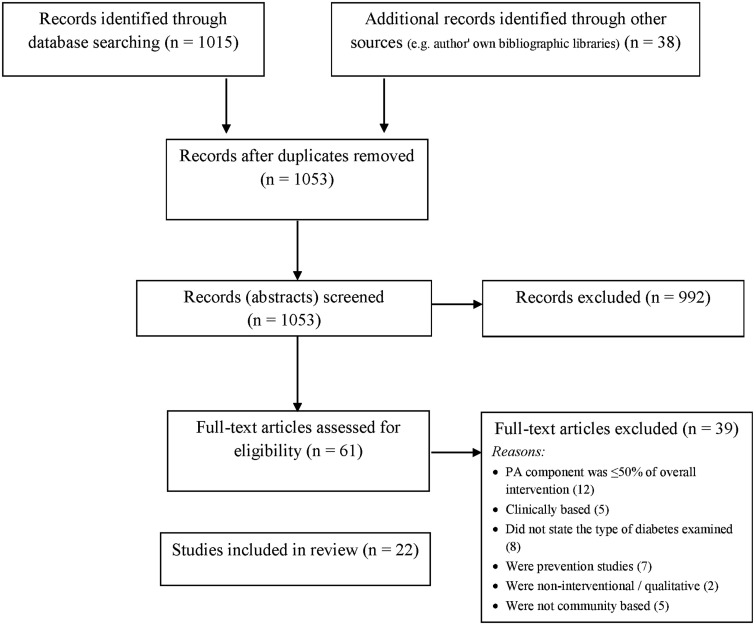
**Flow of study selection through the phases of the review**.

Figure 1 describes the progress through the stages of study selection. The electronic database search strategy provided 1015 references; 38 additional records were identified through other sources. About 1053 records were screened based on titles and abstracts, and of these 992 records were excluded. The remaining 61 records were assessed as full-text articles. Of these 61 studies, 39 did not meet eligibility criteria. The remaining 22 studies were included in our review (see Figure 1).

### Study characteristics

Table 1 reports selected characteristics of all eligible studies. Our search identified 22 studies that examined community-based PA interventions for treatment of T2D, of which a PA component of ≥50% of the overall intervention was employed.

Most studies (16/22, 73%) employed a RCT design. The active intervention periods ranged from 4 weeks (Dutton et al., 2008) to 24 months (Brandon et al., 2003). Study populations ranged from 19 (Martyn-Nemeth et al., 2010) to 652 (Davies et al., 2008). Most of the studies were conducted in the United States (12/20), the remaining studies were conducted in Canada (Mathieu et al., 2008; Plotnikoff et al., 2010, 2011, 2012), Australia (Dunstan et al., 2006; Engel and Lindner, 2006), the United Kingdom (Davies et al., 2008; Skoro-Kondza et al., 2009), Costa Rica (Goldhaber-Fiebert et al., 2003), and Jamaica (Less et al., 2009).

The majority of studies recruited participants from clinics or during visits to general practitioners (14/22), however other community recruitment strategies included local media campaigns (Dunstan et al., 2006; Engel and Lindner, 2006; Klug et al., 2008; Piette et al., 2011; Plotnikoff et al., 2012), intervention open to community (Kruse et al., 2010), and a senior’s center’s (Speer et al., 2008). Four studies employed a combined approach of strategies.

For the purposes of this review, only community-based approaches were considered. The majority of studies (14/22) recruited participants by employing a multi-strategy approach including a combination of the following components: intervention recruitment, delivery, use of facilities, group sessions, and expert advice. In addition studies used of community facilities (Dunstan et al., 2006; Brooks et al., 2007; Davies et al., 2008;, Skoro-Kondza et al., 2009) and expert advice from specialists within the community (Goldhaber-Fiebert et al., 2003; Speer et al., 2008).

Physical activity interventions for treating T2D were eligible only if the PA component/approach accounted for ≥ 50% of the overall intervention. PA approaches including general exercise programs were employed by the majority of studies (9/22), PA counseling/information (Davies et al., 2008; Dutton et al., 2008; Klug et al., 2008; Plotnikoff et al., 2012), walking programs (Goldhaber-Fiebert et al., 2003; Piette et al., 2011), resistance training programs (Dunstan et al., 2006; Brooks et al., 2007; Mathieu et al., 2008; Kruse et al., 2010; Plotnikoff et al., 2011), gym membership provision (Boyd et al., 2006), and yoga classes (Skoro-Kondza et al., 2009).

### Meta-analysis of intervention effects

**Figure 2:**
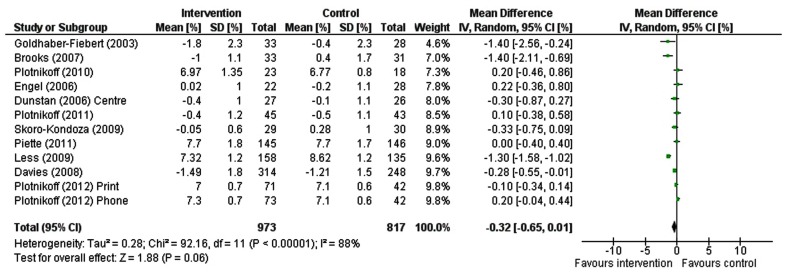
**Forest plot for HbA1c**.

Results from RCTs were pooled to establish the effects of interventions on HbA1c levels (see Figure 2). As there was considerable heterogeneity among interventions [χ^2^ = 92.16, df = 11 (*p* < 0.00001); I^2^ = 88%], the random effects models were used. The impact of interventions on HbA1c levels approached statistical significance (−0.32% (mmol/mol = −26.99) [−0.65, 0.01], Z = 1.88 [*p* < 0.06]).

### Risk of bias

Risk of bias assessment was conducted (see Table 2). One study was identified as low risk of bias (Kruse et al., 2010), 15 studies were classified as moderate risk of bias, the remaining six studies were rated as high risk of bias. Only three studies reported assessor blinding (Item *D*), only four studies presented summary results, estimated effect sizes and precision estimates (Item *G*) and 12 studies employed an objective measure of PA (Item *I*).

**Table 2:** **Methodological quality scores and risk of bias in community-based PA interventions to treat T2D**.

Study	*A* (Separate baseline results)	*B* (randomization)	*C* (dropout rate)	*D* (assessor blinding)	*E* (intention-to-treat)	*F* (confounders)	*G* (effect sizes)	*H* (power)	*I* (objective PA)	Score/9
Plotnikoff et al. (2011)	✓	✓	✓	(?)	✓	✓	×	✓	×	6/9
Piette et al. (2011)	✓	✓	✓	×	✓	✓	×	✓	✓	7/9
Kruse et al. (2010)	✓	✓	✓	✓	✓	✓	✓	✓	✓	9/9
Martyn-Nemeth et al. (2010)	n/a	n/a	✓	n/a	×	×	×	×	×	1/9
Mathieu et al. (2008)	✓	✓	×	×	✓	×	×	×	✓	4/9
Taylor et al. (2009)	✓	✓	×	×	✓	✓	×	✓	✓	6/9
Skoro-Kondza et al. (2009)	✓	✓	(?)	×	✓	(?)	×	×	✓	4/9
Dutton et al. (2008)	✓	✓	(?)	×	✓	✓	×	×	×	4/9
Klug et al. (2008)	n/a	n/a	×	n/a	×	✓	×	×	×	1/9
Speer et al. (2008)	n/a	n/a	×	n/a	×	×	×	✓	×	1/9
Brooks et al. (2007)	✓	×	✓	✓	✓	✓	×	×	✓	6/9
Dunstan et al. (2006)	✓	×	✓	×	✓	✓	×	×	✓	5/9
Boyd et al. (2006)	n/a	n/a	✓	n/a	✓	×	×	×	✓	3/9
Engel and Lindner (2006)	✓	✓	✓	×	×	✓	×	×	×	4/9
Two Feathers et al. (2005)	n/a	n/a	×	n/a	×	✓	×	×	×	1/9
Brandon et al. (2003)	✓	×	×	×	×	×	×	✓	✓	3/9
Goldhaber-Fiebert et al. (2003)	✓	✓	✓	✓	✓	×	×	✓	✓	7/9
Keyserling et al. (2002)	✓	✓	✓	×	✓	✓	×	✓	✓	7/9
Plotnikoff et al., 2010	✓	✓	✓	×	✓	×	✓	✓	✓	7/9
Plotnikoff et al. (2012)	✓	✓	✓	×	×	✓	✓	×	×	5/9
Davies et al. (2008)	✓	✓	×	×	✓	✓	×	×	×	4/9
Less et al. (2009)	✓	×	✓	×	✓	✓	✓	✓	×	6/9

*Criteria: *A*, baseline results reported separately for each group; *B*, randomization clearly described and adequately completed; *C*, dropout ≤20% for ≤6 months follow-up and ≤30% for >6 months follow-up; *D*, assessor blinding; *E*, intention-to-treat analysis; *F*, confounders accounted for in analyses; *G*, summary results presented and estimated effect sizes and precision estimates; *H*, power calculation reported and study adequately powered; *I*, an objective measure of PA was used*.

### Results of included studies

A summary of results is presented in Table 1. A brief description of results comparing intervention to control group effects is presented below.

#### HbA1c

The association between community-based PA interventions and HbA1c was examined in 12 studies (see Table 1). HbA1c improvements were observed in eight studies. Four studies reported no difference in HbA1c levels between intervention and control groups (Plotnikoff et al., 2011, 2012), or between baseline and follow-up (Engel and Lindner, 2006; Piette et al., 2011). Interestingly, in one study, a reduction in HbA1c levels was evident after the intervention phase, however this increased after 6 months (Skoro-Kondza et al., 2009).

#### Weight

Weight outcomes associated with the community-based PA interventions were examined in four studies. Reduction in weight and BMI were observed in three of the four studies. Two studies (Goldhaber-Fiebert et al., 2003; Boyd et al., 2006) reported weight loss in comparison to the control group. While significant reductions in waist circumference and weight were observed in one study (Engel and Lindner, 2006), significant changes in BMI was not evident until 6-month follow-up. One study reported no change in BMI (Martyn-Nemeth et al., 2010).

#### Physical activity

Physical activity was reported in nine studies as a primary outcome. Increases in overall PA was observed (Keyserling et al., 2002; Engel and Lindner, 2006; Speer et al., 2008); specifically increased time spent in leisure time PA (Mathieu et al., 2008), number of participants meeting the PA guidelines (Martyn-Nemeth et al., 2010), increased amount of days engaging in PA (Klug et al., 2008), and increased step counts (Plotnikoff et al., 2012). Two studies (Brooks et al., 2007; Plotnikoff et al., 2011) also found improvements in muscles strength and muscle quality. Three studies reported no change or improvement in PA (Plotnikoff et al., 2012), muscular strength (Taylor et al., 2009), or balance (Kruse et al., 2010).

#### Other outcomes

Other health outcomes included improved quality of life (Mathieu et al., 2008), improved psychological well-being (Martyn-Nemeth et al., 2010), reduced diastolic blood pressure (Boyd et al., 2006; Mathieu et al., 2008), reduced systolic blood pressure (Boyd et al., 2006; Mathieu et al., 2008), improvements in dietary and PA knowledge (Two Feathers et al., 2005), and decreased fasting plasma glucose (Goldhaber-Fiebert et al., 2003).

## Discussion

The objective of this paper was to conduct a systematic review with meta-analysis to investigate the effectiveness of community-based PA interventions for the treatment of T2D in adult populations. Effective treatment/management of T2D can prevent the development of microvascular complications and risk of cardiovascular diseases, which are the leading cause of death in diabetic patients (Stamler et al., 1993; Gilmer et al., 1997). In this review, 22 eligible studies were identified in which the PA component/approach accounted for ≥ 50% of the overall intervention. The studies were conducted across different countries, and represented different ethnic/cultural groups. Although most studies were adequately powered, not all studies provided power calculations. The majority of studies (16/22) employed a RCT design. The primary outcomes varied across the studies; HbA1c, weight and PA were the primary outcomes in 11, 4, and 9 studies, respectively. Overall, only one study had a low risk of bias, and the vast majority of studies had a moderate risk of bias. Overall, this review demonstrates that community-based interventions utilizing a large PA component can be effective in treating T2D in terms of decreasing HbA1c levels, reducing weight (including BMI and waist circumference) and increasing PA levels.

Eleven of the studies that reported HbA1c as an outcome measure were pooled in the meta-analysis. Meta-analysis revealed community-based PA interventions contribute to a lowering of HbA1c by −0.32% (−26.99 mmol/mol) which approached statistical significance effect (*p* = 0.06). The results of the meta-analyses may have indeed been stronger than what has been provided in this report, as the control groups in three of the studies used in the meta-analysis (i.e., Dunstan et al., 2006; Engel and Lindner, 2006; Plotnikoff et al., 2011) received a greater intervention dose than “true controls” receiving no intervention and/or those receiving “standard practice.”

It is interesting to note that in a meta-analyses of 14 clinically based exercise trials in the T2D population, Boulé et al. (2001) reported exercise training decreased HbA1c by −0.66% (−30.71 mmol/mol) which is considered to significantly decrease the clinical risk of diabetes complications in this population. Small changes in HbA1c may still be important from a public health perspective, considering small changes at the individual level can translate to substantial changes within the population if the changes are distributed across the entire target (T2D) population. Health promotion experts advocate that practical, low/minimal intensity interventions that might not have large clinical effects, but can be delivered to large numbers of participants, are more likely to have a broader health impact (Tunis et al., 2003).

A variety of factors may have contributed to the limited change in HbA1c observed in the meta-analysis. A floor effect may have occurred in several studies because individuals may have had well-controlled diabetes at baseline. Some studies employed inclusion criteria, where only individuals with high HbA1c levels at baseline were admitted. The ADAPT study (Plotnikoff et al., 2012), one of the larger studies in this review that reported no impact on HbA1c, had a relatively low baseline level of HbA1c.

Different methods used to recruit individuals into the studies may also have had an impact on outcomes. Several of the studies in this review had participant recruitment in the form of advertising (as employed by 6 of 22 studies). It is possible those who respond to public advertising (e.g., media campaigns) may be a biased group who are more active and take “better care” of themselves. There is evidence that individuals who volunteer to participate in these types of studies are healthier on average compared to those who do not voluntarily participate in studies (Plotnikoff et al., 2008; Rosal et al., 2011).

Adherence to intervention studies may have also played a role. For example, a study conducted by Skoro-Kondza et al. (2009), included in this review, report a very low adherence resulting from motivational barriers to program attendance. Also it is possible that individuals who are most in need of intervention treatment do not adhere to programs. For example, in the ADAPT study, Plotnikoff et al. (2012), found that many dropouts initially had low levels of PA, and it was also individuals most needy of PA interventions that did not participate in the full length of the study. Future research should examine strategies to reach and encourage inactive individuals who are genuinely in need of interventions to participate in research studies.

There was considerable heterogeneity in terms of the different intervention strategies used to promote PA. Some techniques included structured group education programs, yoga classes, telephone counseling, YMBA membership, motivational techniques, providing multi-gym apparatus for home use and the use of lay diabetes facilitators. For adults with T2D, studies have shown better metabolic outcomes when interventions focus exclusively on PA compared to strategies that encompass multiple diabetes care behaviors (Conn et al., 2007). However, this difference was not observed in our review.

Considering that this review examined interventions that used ≥ 50% PA component, other factors of the program besides PA may also have influenced HbA1c levels in participants, such as changes in diet and sitting time. Fourteen of the 22 studies had a dietary component and/or measured dietary changes. However, studies have shown when diet and exercise approaches were combined, the effect on HbA1c was similar to the effect of exercise alone (Boulé et al., 2001). Further, no studies included the promotion of reducing sitting time behavior. Future studies should include strategies to promote the reduction of this sedentary activity given its negative impact on metabolic health (van der Ploeg et al., 2012).

In the set of studies reviewed, five studies (Dunstan et al., 2006; Brooks et al., 2007; Mathieu et al., 2008; Kruse et al., 2010; Plotnikoff et al., 2011) incorporated resistance training mode of activity either as the main focus of the intervention or as part of the intervention. Resistance training has been recognized as a useful therapeutic tool for the treatment of a number of chronic diseases and has been demonstrated as safe and efficacious for elderly and obese individuals (Eves and Plotnikoff, 2006). Resistance training has the potential for increasing muscle strength, lean muscle mass, and bone mineral density, which could enhance functional status and glycemic control.

It is generally accepted that behavior change programs for PA that are theoretically grounded are more efficacious/effective than a theoretical strategies (Biddle et al., 2007). In the reviewed studies, six studies had interventions based in social-cognitive theories, or interventions that were tailored based on theories. Different theoretical frameworks utilized include Social Ecological Model, Health Belief Model, Theory of Planned Behavior, and Social-Cognitive Theory. In this regard, it should be noted that there are limited PA theory-based, long-term studies with large samples targeting the adult T2D population. Few studies have explored the mechanisms of behavior change in community-based interventions for individuals with T2D. One notable exception is the ADHERES study (Plotnikoff et al., 2010) which reported in a secondary analysis that RT planning strategies mediated the effect of the intervention on RT behavior (Lubans et al., 2011). Researchers are encouraged to operationalize their interventions and test the hypothesized mediators of behavior change.

In this review, 10 studies reviewed employed self-report measures for PA, and 12 studies employed objective measures. Although assessing objective measures of PA may not always be feasible in community-based interventions, objective measurement techniques using recent technologies such as accelerometers and pedometers as well as GPS devices wherever possible should be used to assess levels of PA given the over-reporting in self-report PA measures.

In terms of study follow-up, the maximum followed-up time was 2 years (Brandon et al., 2003). In the literature, there is generally limited information on long-term outcomes of interventions, and sharp declines in intervention benefits after a few months have also been observed (Deakin et al., 2005). Future studies need to investigate the effectiveness of interventions to produce long-term change in PA levels, and how effectiveness could be sustained by using strategies such as booster sessions following interventions to facilitate longer-term behavior change.

Considering that the studies included in this review represent a variety of ethnic groups (Mexican, Latino, Caucasian, African-American), a range of age groups (ages 52 to 73), and were 64% female, study findings are perhaps by and large generalizable. However, the variability between the economic and health status of countries included in this review (for example, USA and Canada vs. Jamaica and Costa Rica), must also be considered in the interpretation of the study’s results. In the meta-analysis, we did not conduct additional analyses to investigate intervention features that may moderate intervention effectiveness (e.g., mode of delivery, objective PA vs. self-report PA, age, sex) due to the relatively few number of studies that qualified for the analysis. Further studies which collect such information could be useful to inform the development of future research and interventions. As the number of trials of community-based interventions to increase PA grows, future research should attempt to isolate the impact of specific intervention features on PA change as well as implement high quality study-designs that will allow such investigations.

In terms of a health economical aspect, there is evidence of lower health care utilization and costs in T2D individuals who meet minimum PA guidelines (Plotnikoff et al., 2008). Other studies have shown community-based programs that target PA appear to be cost-effective for individuals with diabetes (Jacobs-van der Bruggen et al., 2007; Roux et al., 2008). Community-based approaches to increase PA have the potential to improve program adherence by using intervention strategies that directly address factors such as socio-economic, psychological, social, and cultural elements (Rosal et al., 2011). Although resource-intensive, clinical approaches can be effective. Future research may consider the combination of clinical and community-based approaches to maximize treatment effects.

Strengths of the study include being the first study, to our knowledge, to meta-analyze solely on community-based interventions to increase PA. Studies representative of a variety of cultural groups from different countries is an additional strength. Limitations include the difficulty in generalizing the contribution of multi-component interventions, and the relatively small number of studies that were included in the meta-analysis. The reviewed studies have moderate levels of risk bias in the methodological quality which should also be considered in the interpretation of these findings. Future community-based trials should be methodologically more rigorous. Publication bias of studies should also be taken into consideration when interpreting the results of this meta-analysis. Studies that do not find statistically significant results may not be published either due to authors’ not attempting to publish or journals not accepting the article for publication, and therefore, this meta-analysis should be interpreted with caution.

Overall, our findings demonstrate community-based PA interventions can be effective for increasing PA and decreasing HbA1c levels. The change in HbA1c was clinically significant in the meta-analysis. Producing even small effects can be meaningful at a ‘population level’ for adults living with T2D. As the number of trials of community-based interventions to increase PA grows in the future, the findings of this meta-analysis will need to be replicated, and intervention features and elements that may moderate intervention effectiveness can be further investigated.

## Conflict of Interest Statement

The authors declare that the research was conducted in the absence of any commercial or financial relationships that could be construed as a potential conflict of interest.
